# The Conjoint Analysis of Microstructural and Morphological Changes of Gray Matter During Aging

**DOI:** 10.3389/fneur.2019.00184

**Published:** 2019-03-12

**Authors:** Xin Zhao, Qiong Wu, Yuanyuan Chen, Xizi Song, Hongyan Ni, Dong Ming

**Affiliations:** ^1^Department of Biomedical Engineering, College of Precision Instruments and Optoelectronics Engineering, Tianjin University, Tianjin, China; ^2^Tianjin International Joint Research Center for Neural Engineering, Academy of Medical Engineering and Translational Medicine, Tianjin University, Tianjin, China; ^3^Department of Radiology, Tianjin First Center Hospital, Tianjin, China

**Keywords:** aging, gray matter, brain atrophy, microstructural degeneration, macro-microstructure associations

## Abstract

Macromorphological and microstructural changes of gray matter (GM) happen during brain normal aging. However, the mechanism of macro-microstructure association is still unclear, which is of guidance for understanding many neurodegenerative diseases. In this study, adopting structural magnetic resonance imaging (sMRI) and diffusion kurtosis imaging (DKI), GM aging pattern was characterized and its macro-microstructure associations were revealed. For 60 subjects among the ages of 47–79, the DKI and T1-weighted images were investigated with voxel-based analysis. The results showed age-related overlapped patterns between morphological and microstructural alterations during normal aging. It was worth noting that morphological changes and mean diffusivity (MD) indexes abnormalities mainly overlapped in the following regions, superior frontal gyrus, inferior frontal gyrus, cingulum gyrus, superior temporal gyrus, insula, and thalamus. Besides, overlapped with GM atrophies, mean kurtosis (MK) abnormalities were observed in superior frontal gyrus, inferior frontal gyrus, transverse temporal gyrus, insula, and thalamus. What important was that intrinsic aging independent associations between macrostructure and microstructure were found especially in media superior frontal gyrus, which revealed the potential mechanisms in the process of aging. The physiological mechanism may be associated with the elimination of neurons and synapses and the shrinkage of large neurons. Understanding the associations of GM volume changes and microstructural changes can account for the underlying mechanisms of aging and age-related neurodegenerative diseases.

## Introduction

Normal aging is an irreversible and uncontrollable process, with a rapid increasing population in recent years. It's a common knowledge that the cognitive function gradually declines during normal aging, including memory (1), language (2), and motor control (3). Although the impact of cognitive function decline on the elderly is not fatal, it does cause inconvenience in the daily life. What' more, many studies have reported that some neurodegenerative diseases occurred easily with advanced age, such as Alzheimer's disease (4), Parkinson's disease (5) and Multiple Sclerosis disease (6). Actually, it's commonly accepted that the function decline is due to the widely degeneration of brain structure, including both microstructural and morphological changes (7–9). Morphological changes mainly refer to extensive gray matter (GM) volume atrophy, which is macroscopical. Many studies have investigated the volume decrease of cortical GM and subcortical GM, revealing a subsequent accelerating decline during normal aging (10–12). Microstructural changes mainly involve the integrity of microstructure for water diffusion, like the permeability of cell membrance or myelinsheath, the concentration of macromoleculars which all greatly support the high efficiency of orderly neuro signal conduction and processing (13–15). Literatures converge that the microstructures will gradually break down with aging procedure (16, 17). Barrick et al. (18) indicated that age-related microstructural alterations in white matter (WM) structure were detected over a relatively short follow-up period and revealed significant changes in WM integrity throughout the brain during normal aging.

The knowledge of either microstructural or macrostructural changes with aging have been well-established. However, several recent studies (19, 20) mentioned the possibly underlying relation between these two kinds of changes. There are no studies involving this issue during aging procedure yet. However, several papers have drawn pictures about the potential associated relations in aging. Tamnes et al. (21) reported regional correlations between cortical thickness and WM microstructure measures across the brain from childhood to adulthood, which directly demonstrated that cortical development to a moderate degree was descriptive of WM development measured by diffusion tensor imaging (DTI). In the aging-related neurodegenerative diseases such as Parkinson's disease, Sterling et al. (19) reported that the volume of cortical GM was negatively correlated with the diffusion indexes of subcortical. Also, evidence has shown that WM changes are considered as the results of Wallerian degeneration, which is secondary to loss of neurons in cortical GM (22). That is, the changes of GM before that of WM at the microscopic level, which deserves more attentions. These findings bring a lot that the underlying associations between micro and macro brain structural degeneration might be a newly deep knowledge about aging.

The development of magnetic resonance imaging (MRI) technology is conducive to exploring the changes of brain internal structure caused by physiological and pathological factors in the development process (23–25). T1 weighted images with high resolution is commonly used with many newly developed algorithms to measure the morphological features like volume, cortical thickness and folding pattern of gyrus (26). Diffusion weighted images are also the basic used to fit some reconstruction models like DTI, high angular resolution diffusion imaging, and diffusion spectrum imaging. Diffusion kurtosis imaging (DKI) was proposed to make up the limitations of DTI with a diffusion measure and a new kurtosis measure, which can indicate the water diffusion restriction and the diffusion heterogeneity in the microstructure (27, 28). Using DKI in Gong's recent study about Alzheimer's disease (29), spatial overlapping and underlying correlation between cortical atrophy and microstructural degeneration associated with Alzheimer's disease were observed. They suggested that this correlation may reflect the chain responses to aging. However, the correlation between brain cortical volume and microstructural changes are not directly measured yet.

In this paper, we utilized technologies mentioned above to access the age-related changes of volume and microstructural properties of GM during normal aging. With voxel-wised analysis and regional based correlation analysis, we accessed the aging related overlapped patterns and associations between this two kinds of changes. We hypothesize that there are regions, of which the volume and microstructure changes significantly correlated, may or may not due to sharing the common patterns with aging. We expect to find some aging-independent regions with significant associations between micro and macro changes of aging brain, which can support that aging is an whole aggressive procedure from micro to macro changes and provide better understanding for human brain aging mechanism.

## Materials and Methods

### Subjects

All MRI data used in this study were obtained from the Tianjin First Central Hospital, China, which was approved by the ethics committee. Every subject has signed written informed consent, which is in accordance with the Human Research Committee guidelines. A total of 60 healthy participants aged 47–79 years (23 Male and 37 Female; mean age 62.2 years, standard deviation, *SD* = 6.8 years) were recruited. All subjects underwent rigorous clinical tests and mental evaluations and found no history of neuropathologic or psychiatric disorders. Moreover, they were examined the Mini-Mental State Examination (MMSE, Chinese version), and there was no significant difference in age between male and female participants (*p* = 0.587). The detailed demographic and neuropsychological information are listed in Table 1.

**Table 1:** Demographic and cognitive characteristics of all participants.

	**Male**	**Female**	***p*-value**
Number	23	37	–
Age (year)	62.8 ± 8.1	61.8 ± 5.8	0.587
Education (year)	12.0 ± 2.9	10.1 ± 3.3	0.022
MMSE	28.9 ± 0.8	29.1 ± 0.7	0.321
CDR	0	0	–

### Magnetic Resonance Images Acquisition

All MRI data were acquired on a 3.0 T MRI scanner (Siemens, Trio) using a 32-channel head coil. T1 and DKI images were collected when the subjects were in a calm and conscious state. Anatomic 3D-T1 images were mainly used as a reference for the anatomical structure and location, and the acquisition parameters were as follows: TR/TE = 1,900/2.5 ms, flip-angle = 9°, lice thickness = 1 mm, voxel size = 1 × 1 × 1mm^3^, matrix = 256 × 256, FOV = 256 × 256 mm, 176 slices. Diffusion weighted (DW) MRI images were acquired for DKI images calculating with the parameters: TR/TE = 10,500/110 ms, slices = 73, voxel size = 1.8 × 1.8 × 1.8 mm^3^, matrix = 128 × 128, FOV = 230 mm × 230 mm, 30 non-collinear diffusion gradient directions with b = 1,000, 2,000 s/mm^2^ and one non-diffusion-weighted scan (b0: b = 0 s/mm^2^).

### Voxel Based Morphometric Analysis

Voxel-based-morphometry (VBM) (30, 31) is an automated technique that has grown in popularity because of the fact that it is relatively easy to use and has provided biologically plausible results. It uses statistics to identify differences in brain anatomy between groups of subjects, which in turn can be used to infer the presence of atrophy. First, all 60 T1-weighted MRI images entered into the same stereotactic space to correct for global brain shape differences, using the International Consortium for Brain Mapping (ICBM) templates. Then, the images were segmented into GM, WM, and cerebrospinal fluid (CSF), and normalized (affine and 16 iteration non-linear transformations) to standard space based on the Montreal Neurological Institute (MNI) template. After that, the normalized and segmented GM images were modulated to correct voxel signal intensity as follows: obtained the transformation parameters based on the registration process, calculated the Jacobian determinants of the deformation field, multiplied the grayscale of each voxel. Image intensities are scaled by the amount of contraction that has occurred during spatial normalization, so that the total amount of GM remains the same as in the original image. The obtained parameters reflected the degree of local deformation of individual anatomical images due to different degrees of GM atrophy. Finally, the images were smoothed using a 12-mm full width at half maximum (FWHM) Gaussian kernel. Smoothing makes the data conform more closely to the Gaussian field model, which is an important assumption of VBM, renders the data more normally distributed, increases the validity of parametric tests, and reduces intersubject variability (32). Data processing was performed using VBM8 (http://www.neuro.uni-jena.de/) and SPM12 (http://www.fil.ion.ucl.ac.uk/spm/) toolbox implemented in Matlab (Mathworks Inc., Natick, MA, USA) platform. Multiple linear regression was performed to define the age-related brain atrophy regions in voxel-wise, treating gender and head motion parameters as confounding variables.

### Voxel Based Analysis of Diffusional Indexes

Diffusion weighted data were processed based on FMRIB Software Library (FSL, https://fsl.fmrib.ox.ac.uk/fsl/fslwiki/) software package (Linux, UK), including distortion correction, eddy-current correction, head motion correction using a 12 degree of freedom FLIRT in reference to b0 images and skull stripping (removing non-brain tissues) in FSL (33–35). Diffusional indexes including mean diffusion (MD) coefficient and mean kurtosis (MK) coefficient were estimated using DKE (diffusion kurtosis estimator, https://www.nitrc.org/projects/dke/) software. Apparent diffusion and kurtosis coefficients were calculated to estimate diffusion tensor and kurtosis tensor according to DKI methods (27, 36), yielding two summary indexes: MD and MK. Then, a two-step of non-linear registration was estimated with b0 images and applied on all diffusional indexes: (1) linearly affined to high-resolution T1 weighted images in native space; (2) non-linear registration from native anatomical images to standard T1 template using the transformation estimated in VBM. All diffusional indexes were finally in a resolution of 2 × 2 × 2 mm. A gaussian kernel with FWHM = 6 mm was then used for spatial smoothing on the MD and MK images before voxel-based analysis in SPM12. Multiple linear regression was applied to find aging related regions, treating gender, and head motion parameters as confounding variables.

### Atlas Based Regional Analysis

The template of automatic atlas labeling (AAL) was used to extract the regional measures of GM for both morphological and microstructural changes. The measures of voxels within each AAL regions were averaged into a mean value. Original correlations (Pearson) between morphological and diffusional measures as well as their partial correlations (Pearson) controlling age effect were accessed.

## Results

### Aging Related Changes in Volume and Diffusional Indexes

**Figure 1:**
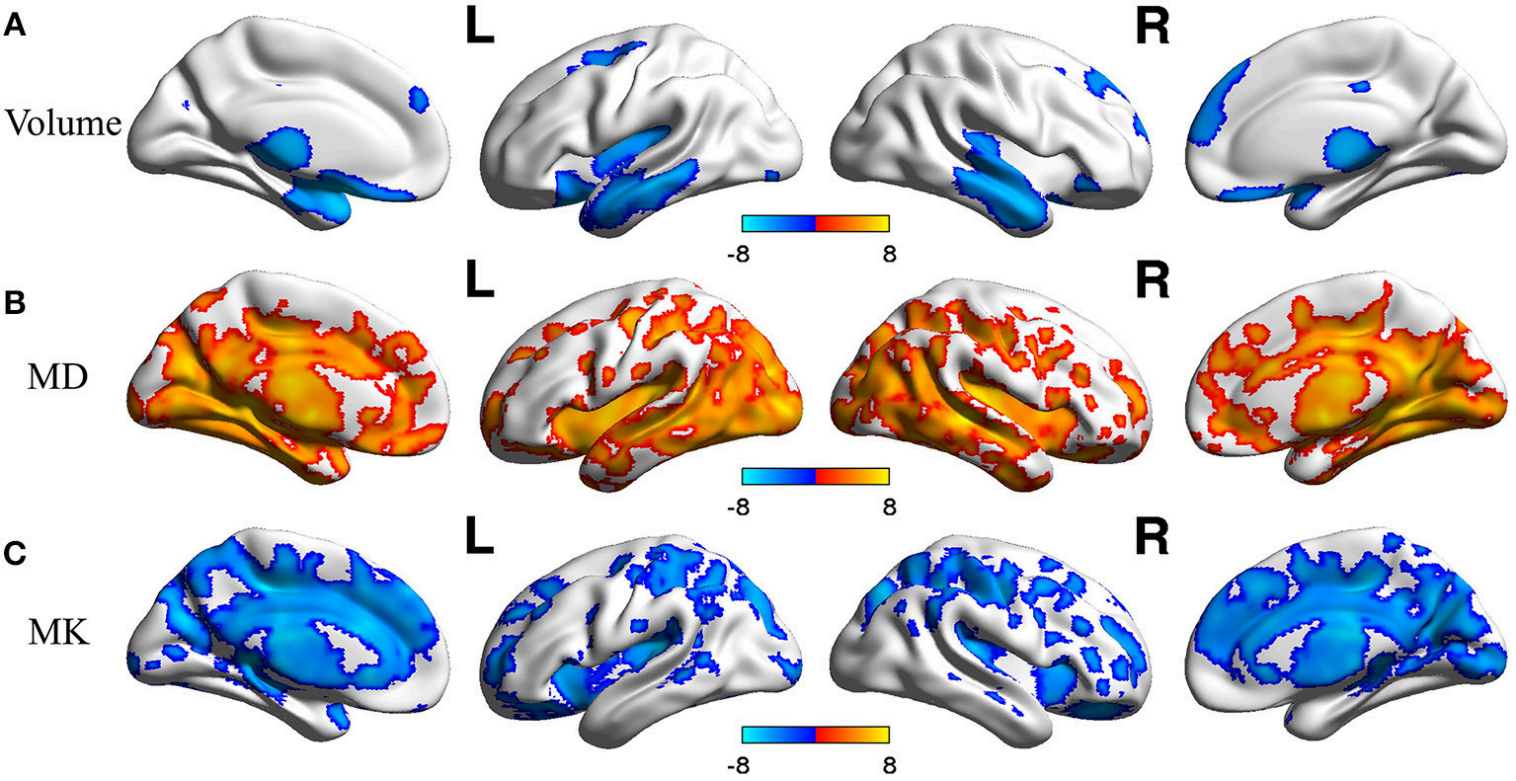
The age-related changes of GM volume and DKI indexes at each voxel. Red color reflects increase in measures with age, and blue color reflects decrease in measures with age. L, left hemisphere; R, right hemisphere. The significant FDR corrected *p*-value < 0.001. **(A)** depicted the age-related changes of GM; **(B)** depicted the age-related changes of MD; **(C)** depicted the age-related changes of MK.

**Figure 2:**
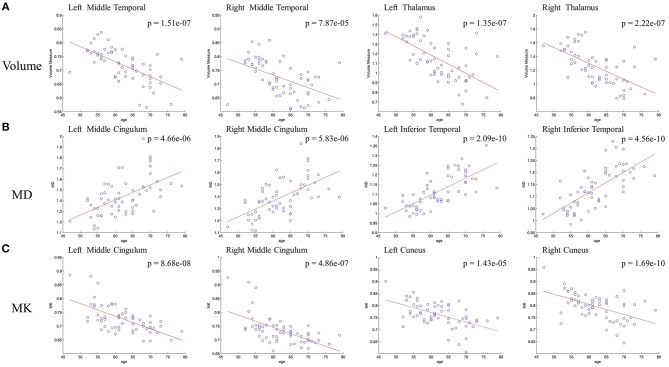
Scatterplots of the linear age effects on **(A)** GM volume and DKI indexes [MD **(B)** and MK **(C)**] in representative regions. Significant decrease were observed between GM volume and age, also between MK and age. Significant increase was observed between MD and age.

In voxel based analysis, a false discovery rate (FDR) corrected *p*-value < 0.001 with cluster size more than 10 was used to define the significant results. Significant negative correlation between the volume of GM and age was observed (Figure 1A), in distinct regions as follow: prefrontal lobe (bilateral superior, middle and inferior frontal gyrus, cingulum gyrus, and olfactory cortex), frontal lobe (left central sulcus), occipital lobe (bilateral lingual gyrus), temporal lobe (most in bilateral middle temporal gyrus, also in bilateral superior and inferior temporal gyrus, fusiform gyrus, amygdala, and left hippocampus, parahippocampal cortex), and subcortical nuclei (bilateral thalamus, insula and left caudate nucleus, putamen). Besides, scatter diagrams were drawn for those significant negative correlation regions to describe the linear relationship between GM volume and age (Figure 2A). Negative linear relationship was detected in bilateral middle temporal gyrus and bilateral thalamus, which showed significant changes in the Figure 1A.

The age-related changes about diffusional indexes were shown in Figures 1B,C. MD increased significantly with age (Figure 1B) almost in all lobes of GM, which mainly included prefrontal lobe (left superior frontal gyrus, inferior frontal gyrus, and anterior cingulum gyrus), frontal lobe (bilateral central sulcus and medial cingulum gyrus), parietal lobe (left precuneus), occipital lobe (bilateral cuneus, lingual gyrus and middle occipital gyrus, inferior occipital gyrus), temporal lobe (bilateral superior, middle and inferior temporal gyrus, fusiform gyrus, transverse temporal gyrus, and left hippocampus), and subcortical nuclei (bilateral thalamus and insula).

Regarding MK (Figure 1C), microstructural changes were observed negatively correlated with age. The areas of significant correlation included prefrontal lobe (left medial and lateral superior frontal gyrus, middle frontal gyrus, left inferior frontal gyrus, and bilateral anterior cingulum gyrus), frontal lobe (left central sulcus and bilateral medial cingulum gyrus), parietal lobe (bilateral precuneus, and inferior parietal gyrus), occipital lobe (bilateral cuneus and left superior occipital gyrus), temporal lobe (bilateral superior temporal gyrus, transverse temporal gyrus, fusiform gyrus and hippocampus), and subcortical nuclei (bilateral thalamus, insula, caudate nucleus, and left putamen). Scatterplots for the linear correlation between DKI indexes and age were shown in Figures 2B,C. There was a positive correlation between MD and age in the bilateral middle cingulum gyrus and bilateral inferior temporal gyrus. A negative correlation between MK and age was observed in the bilateral middle cingulum gyrus and bilateral cuneus.

### Aging Related Overlapped Regions of GM Volume and Diffusion Indexes

**Figure 3:**
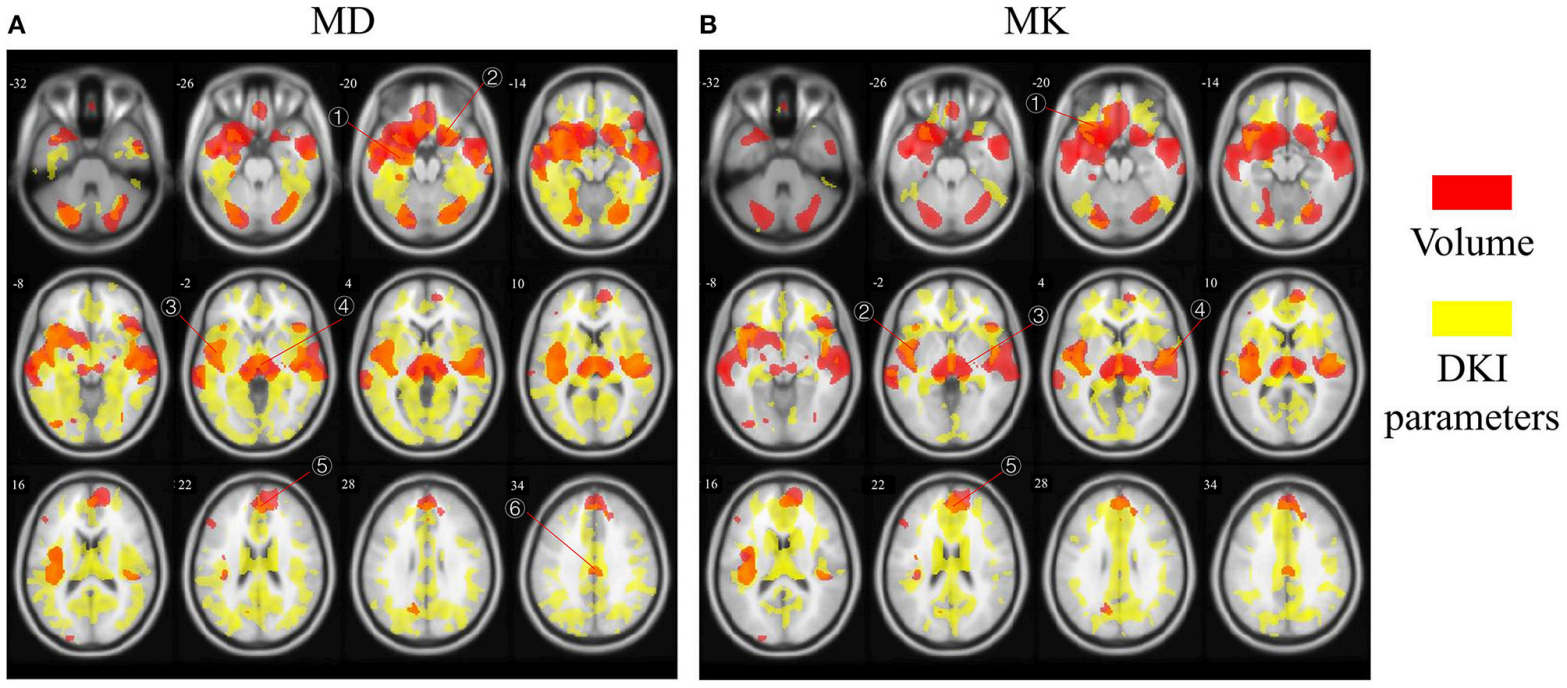
The age-related regions of GM volume overlapped with MD and MK, respectively. Red areas represent GM volume changes, and yellow areas represent microstructural changes. The overlapped regions between **(A)** GM volume and MD and **(B)** GM volume and MK. The labled areas in **(A)** included: right superior temporal gyrus, left inferior frontal gyrus, left insula, thalamus, superior frontal gyrus, cingulum gyrus; in **(B)** included left inferior frontal gyrus, left insula, thalamus, superior frontal gyrus, transverse temporal gyrus.

Figure 3 showed different overlap patterns in the regions of macrostructural and microstructural changes (under *p*-value < 0.001). It was especially worth noting that the changes of GM volume and MD had some spatial overlap, which were located in superior frontal gyrus, inferior frontal gyrus, cingulum gyrus, central sulcus, cuneus, lingual gyrus, precentral gyrus, superior occipital gyrus, inferior occipital gyrus, hippocampus, parahippocampal gyrus, superior temporal gyrus, middle temporal gyrus, fusiform gyrus, insula, and thalamus (Figure 3A). For MK, microstructural changes overlapped with volume changes in many regions, predominantly including superior frontal gyrus, middle frontal gyrus, inferior frontal gyrus, cingulum gyrus, central sulcus, postcentral gyrus, cuneus, superior occipital gyrus, hippocampus, transverse temporal gyrus, superior temporal gyrus, insula and thalamus (Figure 3B). What's more, the macrostructural and microstructural changes also have no spatial overlap in some regions.

### Aging Independent Associations Between Volume and Diffusion Indexes

**Figure 4:**
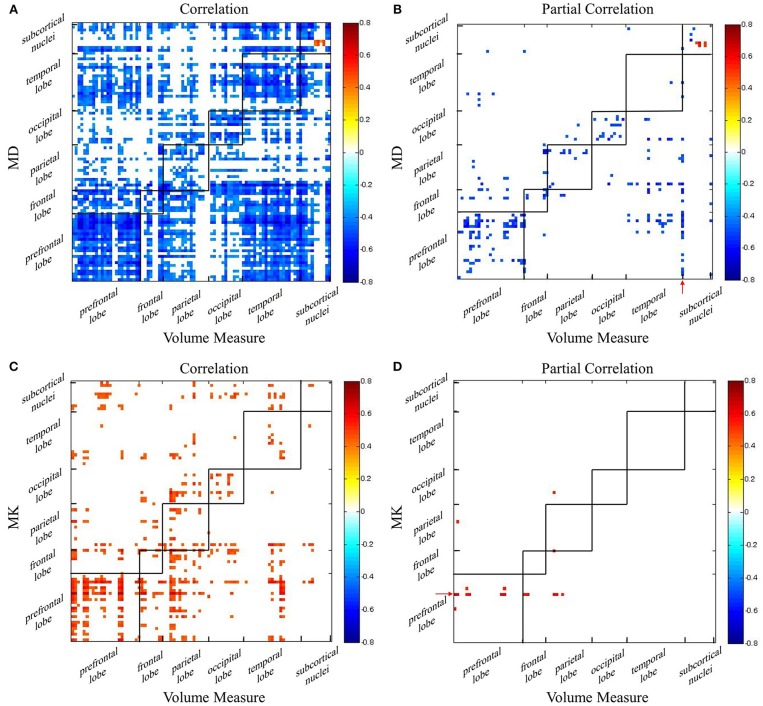
The correlation coefficient maps between GM volume and DKI indexes. Age-dependent association between **(A)** GM volume and MD and **(C)** GM volume and MK. Age-independent association between **(B)** GM volume and MD and **(D)** GM volume and MK. Red color reflects positive correlation, and blue color reflects negative correlation. The significant level is a FDR corrected *p*-value < 0.05.

Using AAL atlas, Pearson correlations and partial Pearson correlations between GM volume and DKI indexes in each region were shown in Figure 4 (FDR corrected *p*-value < 0.05). For MD, we found negative correlations with GM volume across all lobes, and positive correlations only in few areas belonged to subcortical nuclei (Figure 4A). Eliminating the aging factor, the significant correlations between MD and GM volume greatly reduced in the non-diagonal areas. The correlations remained in prefrontal lobe, occipital lobe and subcortical nuclei. Specifically, in the diagonal areas, superior frontal gyrus, middle frontal gyrus, cingulum gyrus, calcarine, and left angular gyrus, precuneus, cuneus, and right lingual gyrus, superior occipital gyrus were observed. What's more, there was a positive correlation between the macrostructural and microstructural changes in putamen (Figure 4B). For most regions, we observed positive correlation between MK and GM volume (Figure 4C). The diagonal correlative regions were mainly located in prefrontal lobe (bilateral medial superior frontal gyrus, right medial frontal gyrus, right inferior frontal gyrus), parietal lobe (right postcentral gyrus, left precuneus), occipital lobe (right cuneus, left superior occipital gyrus) and temporal lobe (bilateral transverse temporal gyrus). After removing the common effects from age, the significant correlation disappeared in almost all regions, except left medial superior frontal gyrus remained in corresponding areas (Figure 4D). It was interesting to note that in the Figure 4B, the volume measure of right inferior temporal gyrus negatively correlated with MD in many regions, like prefrontal lobe, frontal lobe and parietal lobe. Also, there was an age-independent positive correlation between MK in left medial superior frontal gyrus and volume in some regions, such as prefrontal lobe, frontal lobe and parietal lobe (Figure 4D).

**Figure 5:**
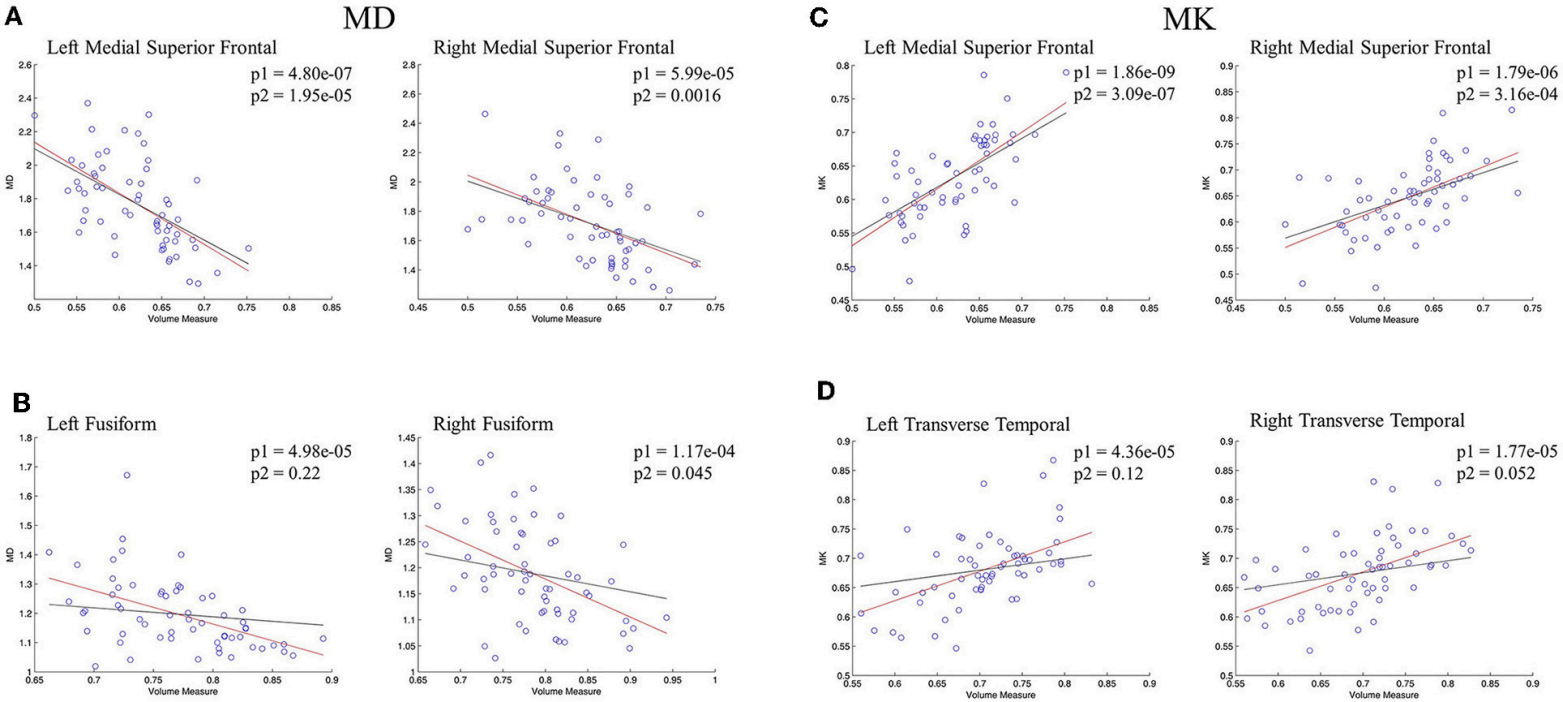
The regression results of GM volume and DKI indexes in representative regions. **(A,B)** showed the scatterplots of GM volume and MD; **(C,D)** showed the scatterplots of GM volume and MK. The red lines represent a linear regression between GM volume and microstructure indexes, and the black lines represents a linear regression between GM volume and microstructure indexes after controlling for age. The significant levels of red lines and black lines were p1 and p2, respectively.

The regional correlations between GM volume and DKI indexes were described further via scatterplots in Figure 5. In bilateral medial superior frontal gyrus, there was a significant negative linear correlation between volume measure and MD, which was still remained after controlling for age (Figure 5A). Significant negative linear correlation between GM volume and MD was also observed in bilateral fusiform gyrus, but, what different was the correlation disappeared without the influence of age (Figure 5B). For MK, significant positive correlation with volume measure was characterized in bilateral medial superior frontal gyrus, whose pattern were similar with MD (Figure 5C). In bilateral transverse temporal gyrus, no significant positive linear correlations between kurtosis measures and GM volume existed after the control of the age (Figure 5D). The regional results conformed to the correlations and partial correlations coefficient maps.

## Discussion

In this study, we firstly generated the aging patterns of microstructural changes and morphological alterations evaluated with diffusional and volume measures in voxel level separately. Then, spatial overlapping and partial correlation were used to describe the association between the two aspects about structural degeneration during aging. To our knowledge, it is the first to characterize the associations between microstructural and morphological changes in the progression of normal aging. The results can be summarized into two aspects: (1) the morphological and microstructural measures significantly altered with aging and overlapped in some regions especially bilateral medial superior frontal gyrus. (2) The morphological and microstructural measures were highly associated with each other independent from aging. For better knowledge about it, discussions were made in detail.

### Aging Related Changes in Morphological and Microstructural Changes

Predominantly age-related decline in the GM volume of prefrontal lobe, temporal lobe, insula, and thalamus are captured, which is in line with the previous study (10, 37–39). The morphological atrophy is consistent with the functional decline that humans exhibit during aging. The previous literature reported that prefrontal lobe showed earlier decline in cognitive functions, which included optional movement and higher order psychological activity with intelligence (40). Several studies (37, 41) indicated that the cognitive processes mediated by the prefrontal lobe were impaired during the normal aging processes. The thalamus plays an important role in sensory nerve conduction process (42). To explain the decrease of GM volume, several studies about the GM physiological structure have been reported (43, 44). They proposed that the reduction in number of synapses and glial cells should mainly account for the reduction of GM volume.

Our results about the diffusion and kurtosis indexes are consistent with the previous articles also showing MD increase and MK decrease during normal aging (45, 46). Das and colleagues (47) reported that, compared with young group, the age-related changes of MD and MK were observed in almost all lobes and subcortical nuclei in elderly group. It is reported that neurodegenerative changes of GM might result in an increase in MD, such as neuron loss, reduced synaptic density, or reduced metabolic activity (48, 49). Literatures suggested MK variation depending on anatomical location as well as age dependency (50–53). The decrease in GM kurtosis index may indicate an increase of cell membrane permeability, demyelination, and decrease in cell packing density (51, 54).

### Overlapping of Aging Related Morphological and Microstructural Changes

Amyloid deposition has been reported to appear in the prefrontal lobe and temporal lobe in the progression of aging (55–57). However, GM regions have received much less attention than WM from MRI studies. The spatial overlapping characteristics of regions between the macrostructure and microstructure in GM are also unclear. In one study of Alzheimer's disease, Wang et al. have captured the changes both volume and MD in subcortical nuclei (58). Our finding of the significant GM volume decrease and MD increase in insula and thalamus. The insula subserves a wide variety of functions in humans ranging from sensory and affective processing to high-level cognition (59). In normal aging, Shafto et al. (60) measured GM density at each voxel in insula. Negative correlations of age with voxel-wise GM density gave a measure of the degree and distribution of age-related atrophy and less hindrance for water molecules at a micro level. The thalamus has a complicated connections with its cortical, subcortical, and cerebellum (61–63). Also, it is a critical node in networks supporting cognitive functions known to decline in normal aging, including component processes of memory and executive functions of attention and information processing (64). Fama et al. (65) reviewed regional thalamic volume shrinkage and microstructural degradation during aging. In addition to insula and thalamus, macrostructure and microstructure alterations occurred simultaneously in prefrontal lobe, frontal lobe, temporal lobe. These areas are responsible for language, movement, auditory sense and vision, which show obvious function decline with advanced age are composed of a variety of neurons, glia and a large number of nerve fibers in and out of the cortex. In the process of aging, loss of nerve cells and disintegration of axons led to loss of microstructural complexity and increase in extracellular free diffusion space (29). These microstructural changes further manifest as increases in MD, decreases in MK. At the same time, large-scale loss of neuronal complexity results in shrinkage that will later condense the structure, which is shown as the morphological atrophy of volume in cortical GM. The physiological mechanisms of these regions need to be further explored.

Except for the overlapping regions, there many regions show different distribution without overlap. Different physiological mechanisms may lead to changes in microstructural indexes and GM volume decrease, that will present inconsistent aging related regions. In the Figure 3, microstructural changes in brain regions are clearly more than volume atrophies. We hypothesize that it is due to the differences on time scales, that is, microstructural changes precedes morphological alterations. This finding is consistent with the results in normal elderly and age-related neurodegenerative diseases (29). Besides, It can be speculated that the dominant decline of GM volume may be caused by the accumulation of microstructure degradation to a certain extent. Therefore, we hypothesize that there should be some correlations between macro-microstructure characteristics.

### Aging Independent Associations Between Morphological and Microstructural Changes

As shown in Figures 4, 5, regional correlations between GM volume and diffusion index and kurtosis index are captured, respectively. What's more, the intrinsic associations between volume measure and microstructural indexes are described in the partial correlation coefficient maps. After eliminating the effect of age, it is obvious that the correlations disappear, especially in non-diagonal regions of GM. The results suggest that associated changes between GM volume and DKI indexes can conceivably be expected to occur as a consequence of age-dependent regional development.

The intrinsic associations in the diagonal regions deserve more attentions. It may be explained by changes of physiological structure at the cellular level. There are many studies have reported that neuron loss is considered as the measure of brain integrity (66–68), including prefrontal lobe which is consistent with our findings. There is also evidence that cell body shrinkage results in the decrease of GM volume (69). During normal aging, the elimination of neurons and synapses and the shrinkage of large neurons are detected to lead to the age-related microstructural changes (69–72). In this process, the atrophy of GM is captured. At the same time, the free diffusion movement of water molecules is less hindered, which is reflected as the increase of MD. Also, as the disappearance of neurons and increase of cell membrane permeability, the environment for the diffusion movement of water molecules is simpler, and the movement of water molecules is closer to the gaussian distribution, which is represented as the decrease of MK. Thus, the different patterns of intrinsic macro-microstructural relationships may be related to the physiological changes in GM, which needs further study. Specially, there is a positive correlation between the volume measure and diffusion index in putamen (Figure 4B). The microstructural compositions of the putamen are relatively simple, which is predominantly constituted by neurons and glia cells (73–75). Aging-related iron deposition is observed in putamen, which may impose extensive hindrance to free diffusion of water molecules (76). The high level of iron content may be a reason for the positive correlation between volume and MD in putamen. Another probable interpretation is that there is no significant decline in the small-cell density with aging in putamen, while the volume decrease (75).

It is interesting to note that, in addition to the diagonal regions, the correlations are acquired in the non-diagonal regions after controlling age. We speculate that there may be regional distribution heterogeneity between these regions. On the one hand, the volume of right inferior temporal gyrus is related with the MD in multiple regions from different lobes (Figure 4B). It may be explained that, in polymodal regions, varied regions such as parietal, temporal, and other frontal cortices were associated by commissural fibers (77). The literature demonstrated that connections between temporal and frontal regions were detected (78–80). As shown in Figure 4D, that MK in left medial superior frontal having a positive with volume of several areas also can be explained. On the other hand, different regions in same lobe show a negative correlation between GM volume and MD (Figure 4B). A possible explanation is that there are spatial and functional connections between adjacent areas.

## Limitations

The present study has several limitations with the need for further attention. First, the same study should be carried out in longitudinal designs. Based on previous assumptions as well as current results, the biological process of GM atrophy and GM microstructural degeneration happen in different time sequence. Investigations on GM volume and microstructure development in the same sample with a wide range of age, can reveal better dynamic changes in the brain over time during aging. The second is the relatively small sample size. To further demonstrate our results, a larger cohorts study should be performed, which includes subjects of all stages in brain aging. What's more, the microstructural changes in diffusivity and kurtosis indexes are captured by DKI. However, DKI model are not sensitive to the complicated compositions like crossing fibers. Finally, more effective statistical analyses to investigate the relationship of morphological measurements and DKI indexes should be used. The effects of gender differences and laterality effects should be studied further.

## Conclusion

To sum up, we revealed the age-independent and intrinsic associations between macrostructure and microstructure in the process of aging. The results demonstrate a possible intrinsic recession mechanisms during normal aging, including the elimination of neurons and synapses, the shrinkage of large neurons. The present study not only strengthen our understanding of potential mechanisms for normal aging, but also lay the foundation for research of neurodegenerative diseases, such as Alzheimer's disease and Parkinson's disease. Considering morphological and microstructural changes usually occur earlier than apparent clinical symptoms, such as cognitive decline and movement disorder, our results is meaningful to detect and prevent abnormal aging.

## Data Availability

All datasets generated for this study are included in the manuscript and/or the supplementary files.

## Ethics Statement

This study was carried out in accordance with the recommendations of Human Research Committee guidelines, Tianjin First Central Hospital ethics committee with written informed consent from all subjects. All subjects gave written informed consent in accordance with the Declaration of Helsinki. The protocol was approved by the Tianjin First Central Hospital ethics committee.

## Author Contributions

QW: data processing and analysis, manuscript writing, and revision. YC: original idea design, data gathering, processing and analysis, provided useful guidance and ideas, and manuscript revision. XS: manuscript revision, provided useful guidance, ideas and research sponsor. HN: data gathering, manuscript revision, provided useful guidance, ideas and research sponsor. XZ and DM: project design and guidance, manuscript review and revision, and provided research sponsor.

### Conflict of Interest Statement

The authors declare that the research was conducted in the absence of any commercial or financial relationships that could be construed as a potential conflict of interest.
